# Loss of PAX8 in high-grade serous ovarian cancer reduces cell survival despite unique modes of action in the fallopian tube and ovarian surface epithelium

**DOI:** 10.18632/oncotarget.9051

**Published:** 2016-04-27

**Authors:** Laura H. Rodgers, Eoghainín Ó hAinmhire, Alexandria N. Young, Joanna E. Burdette

**Affiliations:** ^1^ Department of Medicinal Chemistry and Pharmacognosy, Center for Pharmaceutical Biotechnology, College of Pharmacy, University of Illinois at Chicago, Chicago, IL

**Keywords:** PAX8, fallopian tube, ovary, high grade serous carcinoma (HGSC), FOXM1

## Abstract

High-grade serous carcinoma (HGSC) is the most common and lethal form of ovarian cancer. PAX8 is a transcription factor expressed in fallopian tube epithelial cells and in 80–96% of HGSC tumors. The ovarian surface epithelium (OSE) only acquires PAX8 expression after malignant transformation. In this study, forced PAX8 expression in OSE cells increased proliferation and migration through upregulation of EMT factors such as N-cadherin and Fibronectin. OSE cells expressing PAX8 also had an increase in the FOXM1 pathway, but PAX8 alone was not sufficient to drive tumorigenesis. PAX8 knockdown in the oviductal epithelium cells did not decrease expression of the FOXM1 pathway and induced only a slight decrease in cell proliferation. No changes in migration, cell cycle, or apoptosis were detected after PAX8 knockdown in oviductal cells. Finally, PAX8 knockdown in HGSC cell lines resulted in increased apoptosis and decreased FOXM1 levels. The results presented here suggest that PAX8 has a cell specific role in governing proliferation and migration in nontransformed ovarian surface epithelium cells compared to the oviductal cells, but its reduction in serous cancer cell lines provides a common mechanism for reducing cell survival.

## INTRODUCTION

Ovarian cancer is the most lethal gynecological disease in the United States and the fifth leading cause of cancer-related death in women [1]. High-grade serous carcinoma (HGSC) is the most common and lethal histotype of ovarian cancer. This high mortality rate is due in large part to poor early detection methods leading to late diagnosis after the disease has metastasized [2, 3]. Ambiguity surrounding the progenitor cells of ovarian cancer hinders early diagnosis of this disease. Previously, ovarian cancer was believed to originate solely from the ovarian surface epithelium (OSE) but current research suggests the fallopian tube fimbria can also act as the source for HGSC. Ovarian cancer, therefore, can be considered an umbrella term for a heterogeneous disease encompassing tumors arising from either the fallopian tube or the ovary. Deciphering the cell of origin of a patient's tumor is critical because this knowledge has clinical and research implications. Comparing tumor profiles to “normal” tissue in the OSE has elucidated pathways and genes termed dysfunctional or overexpressed in HGSC. For HGSC originating from the fallopian tube epithelium (FTE), however, these overexpressed proteins and dysfunctional pathways may actually constitute normal FTE expression.

Paired box transcription factor 8 (PAX8) may provide clues regarding a tumor's cell of origin due to its differential expression in the FTE, OSE, and HGSC. During embryogenesis PAX8 is expressed in the Müllerian duct and it continues to be expressed in the secretory cells of the adult FTE [4, 5]. The function of persistent PAX8 in the adult fallopian tube remains unknown. The OSE does not express PAX8 during development, but conflicting reports suggest PAX8 may be acquired in the adult OSE [5, 6]. Ozcan and colleagues suggest the adult ovarian surface cells may contain a heterogeneous group of mesothelial cells negative for PAX8 expression and Müllerian derived cells positive for PAX8 expression [6]. In normal murine OSE, however, PAX8 has never been reported. HGSC cells express PAX8 in 80–96% of cases [6–11]. In fact, pathologists use PAX8 staining as a defining marker of HGSC [12]. The absence of PAX8 in the OSE, coupled with the presence of PAX8 in the FTE and HGSC, might suggest the FTE is the source of HGSC. However, there are multiple models of HGSC derived from the OSE that express PAX8, including a serially passaged OSE cell line and an OSE model with *LKB1* and *PTEN* deletion [13, 14].

In addition to its expression in HGSC, PAX8 is associated with neoplasms of the kidney and thyroid. In thyroid carcinomas, PAX8 undergoes translocation with the PPARγ to create a fusion protein [15]. This fusion protein can act as an oncogene, and is found in approximately 35% of follicular thyroid carcinomas [15]. In rat thyroid epithelial cells, PAX8 increased cell survival and proliferation through transcriptional inhibition of the p53 positive regulator protein, p53inp1 [16]. Knockdown of PAX8 in these epithelial cells induced p53-mediated apoptosis [16]. In renal cell carcinomas (RCC), PAX8 promotes tumor growth through regulation of the E2F1-RB pathway [17]. Knockdown of PAX8 in RCC cell lines led to apoptosis through G_1_/S phase cell cycle arrest. PAX8 directly activated E2F1 transcription by forming a complex with RB protein on the promoter of E2F1 to drive proliferation [17]. These data indicate that PAX8 has a critical role in cell cycle regulation and tumor survival. Despite its ubiquitous expression and role in other tumor types, little is known about what PAX8 regulates in HGSC. Previous research has shown that PAX8 knockdown in HGSC leads to apoptosis as well as an increase in migration, anchorage independent growth, and tumor suppression [18, 19]. The pathways involved in these phenotypic changes, however, remain unknown. In addition, the role of PAX8 in normal fallopian tube cells has not been reported.

This study used three human HGSC cell lines to analyze the pathways downstream of PAX8 in tumorigenic cells. The role of PAX8 in murine oviductal epithelial cells (MOE) and murine ovarian surface epithelium (MOSE) was compared to HGSC to elucidate the function if PAX8 in non-transformed cells of distinct cellular origin. Murine cells were used instead of human cells to answer this question because murine cells are not immortalized with SV40 and therefore have wildtype p53 and retinoblastoma (RB) protein. Characterizing the function of PAX8 in non-transformed FTE and OSE allowed for comparison of PAX8 in HGSC originating from the FTE compared to HGSC originating from the OSE. This knowledge may help clinicians decipher the cell of origin of a patient's cancer and allow for targeted therapy. In addition, these mechanisms may differ between OSE and FTE derived tumors and may be essential when targeting PAX8 in high-grade serous tumors.

## RESULTS

### PAX8 drives proliferation, migration, and EMT in murine OSE cells

**Figure 1:**
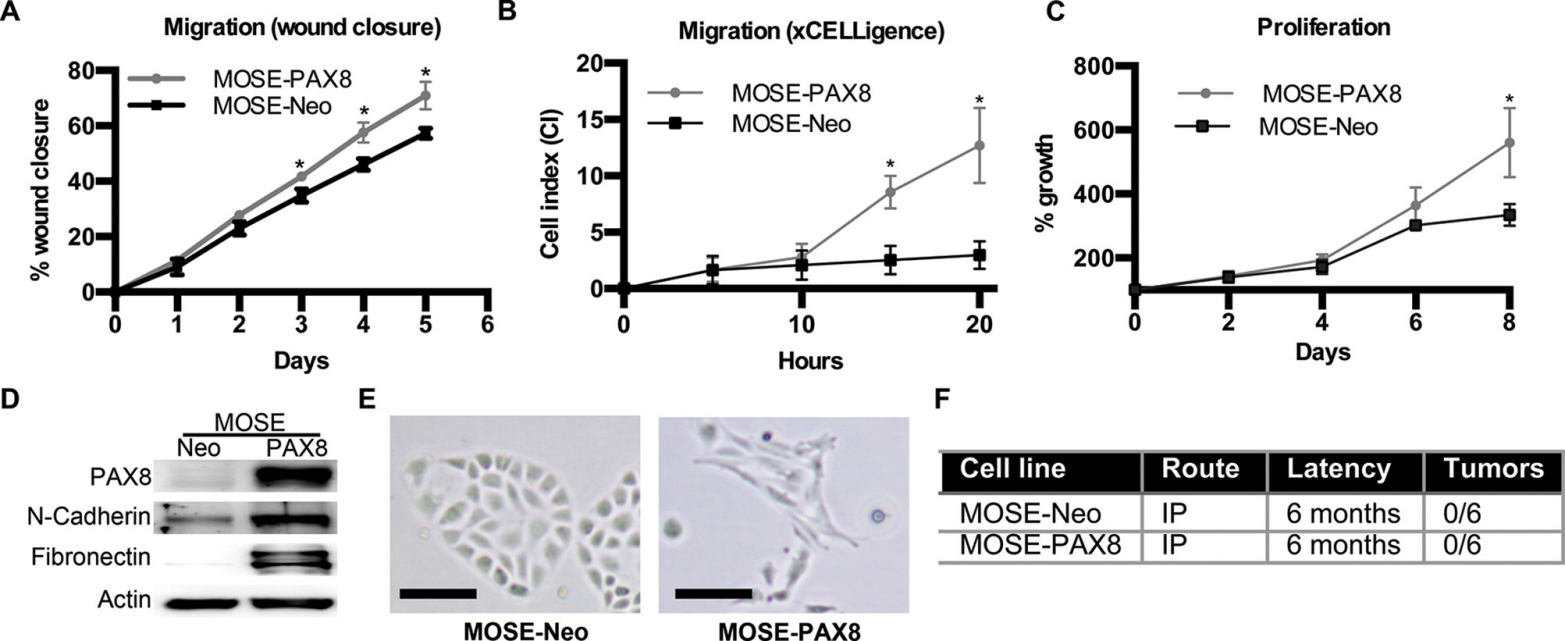
PAX8 expression in murine OSE cells increases migration and proliferation (**A**) Murine ovarian surface epithelium (MOSE) cells with forced PAX8 expression were significantly faster at healing a wound after 3 days (*n* = 3). (**B**) MOSE-PAX8 cells have increased migration through a Boyden chamber as depicted through the xCELLegence assay^®^ (*n* = 3) in 20 hours. (**C**) No change in migration over a wound after 2 days in MOE^siPAX8^ cells (*n* = 4). (**D**) Representative western blot demonstrating increased expression of N-cadherin and Fibronectin after PAX8 expression in MOSE cells. (**E**) MOSE-PAX8 cells have a more mesenchymal, elongated morphology when compared to MOSE-Neo. (**F**) MOSE-NEO and MOSE-PAX8 cells (1 × 10^7^ cells/mouse) were intraperitoneally (IP) injected into nude mice, and internal gross anatomy was examined for tumors after 6 months. Data represent mean ± SD. Significance is represented by * for *p* ≤ 0.05.

The murine OSE (MOSE) does not endogenously express PAX8, yet there are several OSE-derived serous ovarian cancer models that acquire PAX8 expression [13, 14]. To determine if forced expression of PAX8 in the OSE is a component of tumor formation, PAX8 was stably expressed in MOSE cells using a constituently active promoter (MOSE-PAX8). Expression of PAX8 in MOSE cells increased wound closure and migration, suggesting an increase in motility (Figure 1A–1B). MOSE-PAX8 cells also showed an increase in proliferation after 8 days (Figure 1C). Two pro-migratory genes were selected for analysis to verify increased migration. Loss of E-Cadherin and increased N-Cadherin are associated with increased migration and EMT [20]. E-cadherin was not tested in this system as OSE cells lack expression of E-cadherin [20]. Fibronectin is associated with both EMT and migration, and was analyzed by Di Palma and colleagues in their study of PAX8 in SV40 immortalized human IOSE 80 cells [19]. N-cadherin and Fibronectin protein levels were significantly increased in MOSE-PAX8 cells compared to MOSE-Neo control (Figure 1D). There was a 2.0 ± 0.44 mean fold increase in N-Cadherin and 3.8 ± 1.1 mean fold increase in Fibronectin mRNA levels. Compared to MOSE-Neo, the morphology of MOSE-PAX8 cells was altered to a more mesenchymal or elongated morphology (Figure 1E). Anchorage independent growth was not increased by PAX8 expression, suggesting that cells had not undergone neoplastic transformation (Supplementary Figure S1). To confirm that PAX8 is not sufficient to form tumors in MOSE cells, 1 × 10^7^ cells were injected intraperitoneal into 6 mice for 6 months. No tumors were found after dissection (Figure 1F). Thus MOSE-PAX8 induced functional changes such as proliferation and migration, but was not sufficient to cause transformation.

### PAX8 increases expression of the FOXM1 pathway in MOSE

**Figure 2:**
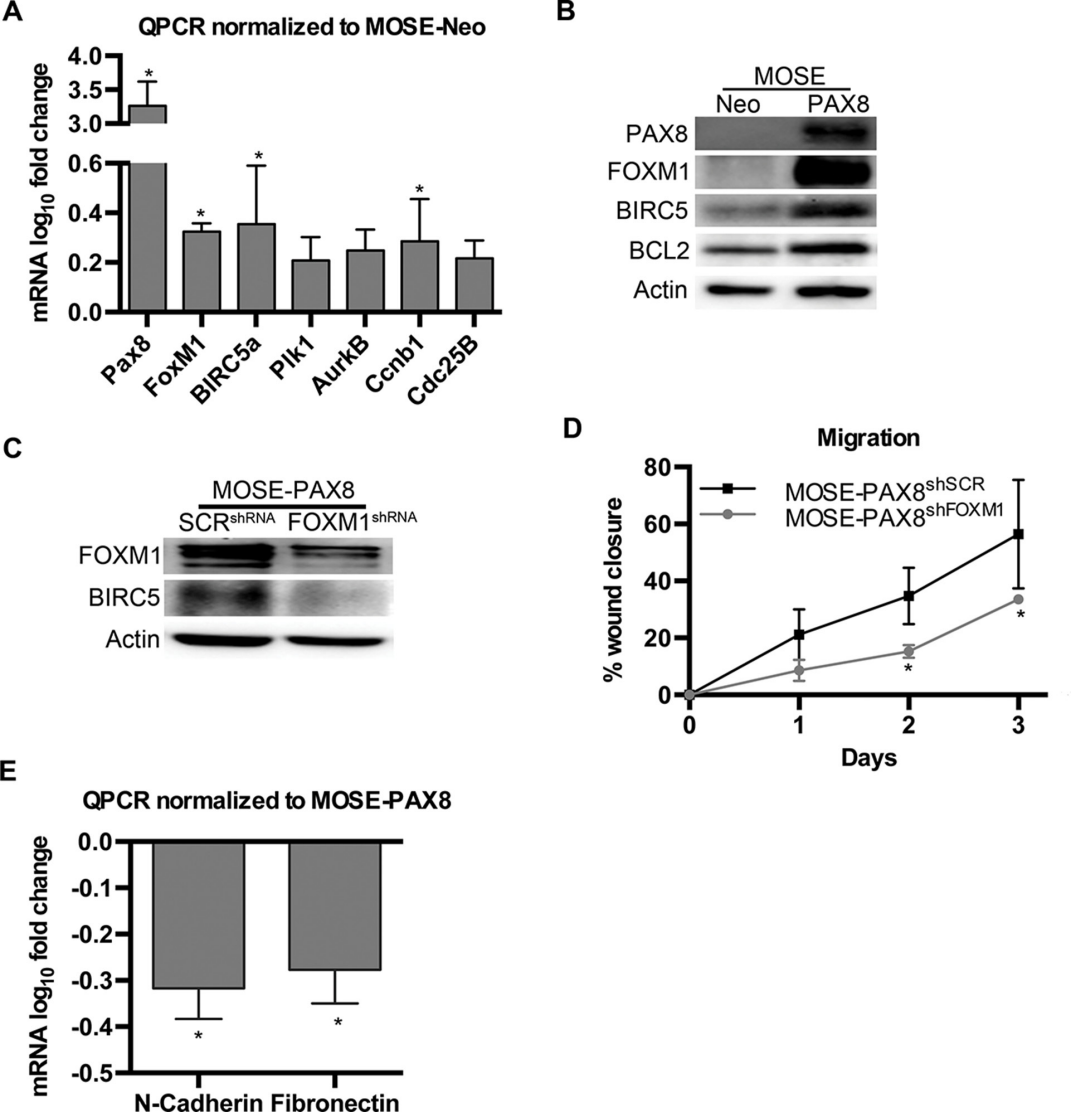
PAX8 expression in MOSE cells increases the FOXM1 pathway (**A**) mRNA levels (log_10_ fold change) of genes in the FOXM1 pathway are significantly increased after PAX8 expression in MOSE cells. Data was normalized to MOSE-Neo control expression levels (*n* = 4). (**B**) Protein levels of genes in the FOXM1 pathway are increased after PAX8 expression in MOSE. (**C**) Western blot confirming a decrease in the FOXM1 pathway after MOSE-PAX8 transfection with FOXM1^shRNA^. (**D**) MOSE-PAX8 cells with knockdown of FOXM1 were significantly less migratory over a wound after 2 days (*n* = 3) when compared to MOSE-PAX8 cells. (**E**) MOSE-PAX8^shFOXM1^ have decreased mRNA levels (log_10_ fold change) of EMT markers (*n* = 4). Data represent mean ± SD. Significance is represented by * for *p* ≤ 0.05.

Previous research has shown PAX8 can bind to the first exon of the p53 gene and inhibit transcription [21]. A separate study reported that wild type p53 decreases expression of FOXM1 [22]. Since the The Cancer Genome Atlas (TCGA) highlights the FOXM1 pathway as activated in 84% of patient samples [23], this study measured the FOXM1 mRNA and protein concentrations in MOSE cells to determine if this pathway is relevant in non-transformed OSE cells with forced PAX8 expression. PAX8 overexpression in these cells led to a significant increase in FOXM1 mRNA as well as some of its downstream targets including BIRC5a and CCNB1 (Figure 2A). There was also an increase in the protein levels of FOXM1, BIRC5a and BCL2 (Figure 2B). BCL2 is a canonical downstream target of PAX8 that served as a positive control for PAX8 activity [24].

To determine if the MOSE-PAX8 proliferative and pro-migratory phenotype was due to FOXM1 expression, MOSE-PAX8^shFOXM1^ cells were generated (Figure 2C). MOSE-PAX8^shFOXM1^ cells had decreased migration compared to MOSE-PAX8 (Figure 2D). N-cadherin and Fibronectin mRNA also decreased in MOSE-PAX8^shFOXM1^ compared to MOSE-PAX8 cells (Figure 2E). There was no change in proliferation after transient FOXM1 knockdown (data not shown). Transient transfection, however, cannot be maintained for the eight days required for the growth differences seen in MOSE-PAX8 compared to MOSE-Neo. Overall these findings suggest the FOXM1 pathway is downstream of PAX8 and contributes to enhanced migration in MOSE-PAX8 cells.

### Silencing PAX8 in MOE cells has minimal functional effects

**Figure 3:**
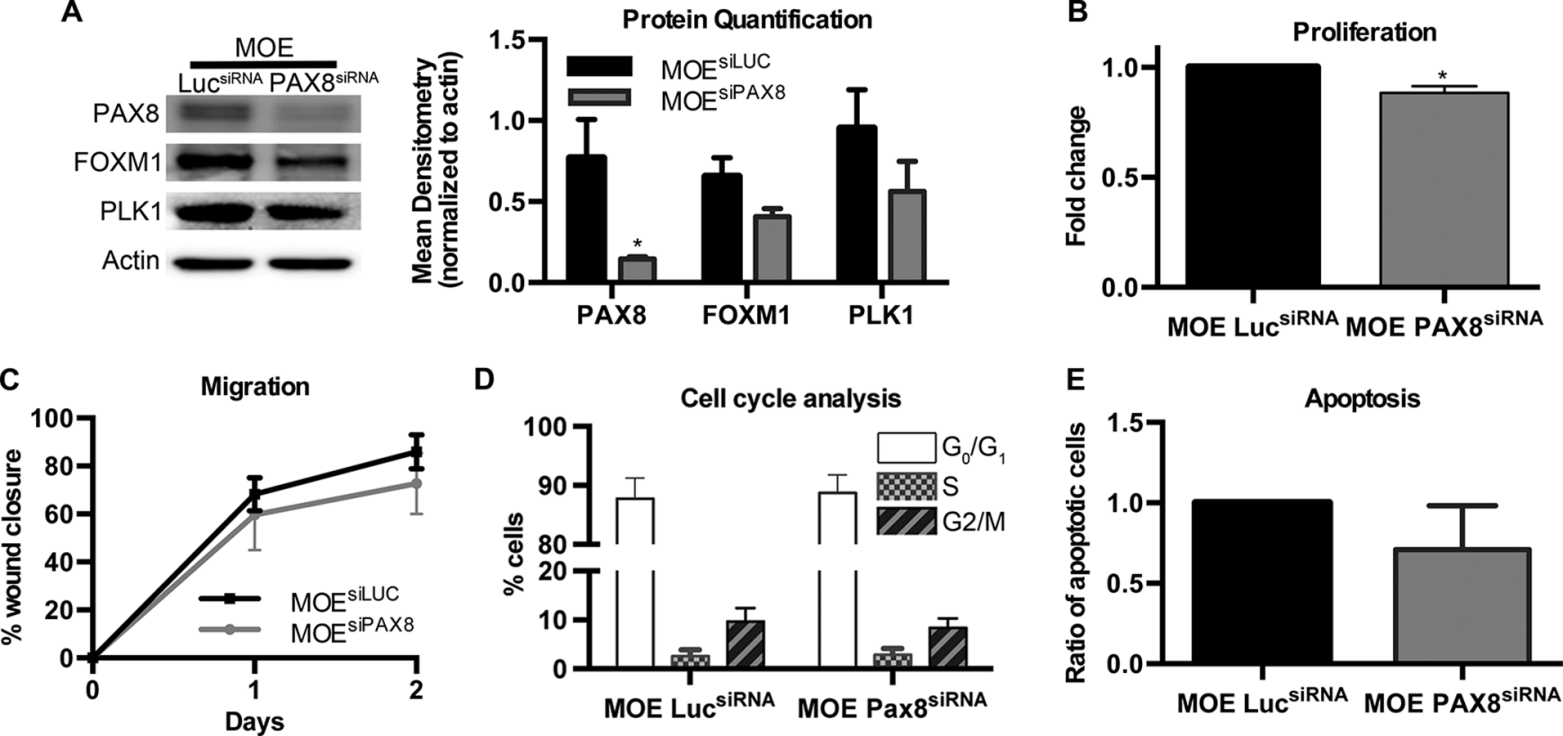
Silencing PAX8 in MOE cells has minimal functional effects (**A**) Protein levels of FOXM1 and the downstream gene PLK1 were not altered after silencing PAX8 in murine oviductal epithelial (MOE) cells. (**B**) SRB assay indicates no change in proliferation in MOE cells with PAX8 silencing (*n* = 3) after 2 days. (**C**) No change in migration over a wound after 2 days in MOE^siPAX8^ cells (*n* = 4). (**D**) No change in cell cycle based on G_o_/G_1_, S, or G_2_/M phase staining (*n* = 3) in MOE^siPAX8^ cells. (**E**) Flow cytometry with Annexin V, APC and PI staining indicates no change in apoptosis after PAX8 silencing (*n* = 3). Data represent mean ± SD. Significance is represented by * for *p* ≤ 0.05.

To determine whether PAX8 has a similar role in MOE cells, where the transcription factor is normally expressed at high levels, siRNA was used to silence PAX8 in MOE cells. MOE^siPAX8^ cells did not demonstrate a significant decrease in the FOXM1 pathway compared to the control MOE^siLUC^ (Figure 3A). PLK1 protein was measured as a downstream marker of FOXM1 because MOE cells do not express high enough amounts of BIRC5a to appear on western blot (Figure 3A). Functional assays showed that despite a slight, but significant decrease in proliferation in MOE^siPAX8^ cells (Figure 3B) there were no changes in migration, cell cycle, or apoptosis (Figure 3C–3E).

**Figure 4:**
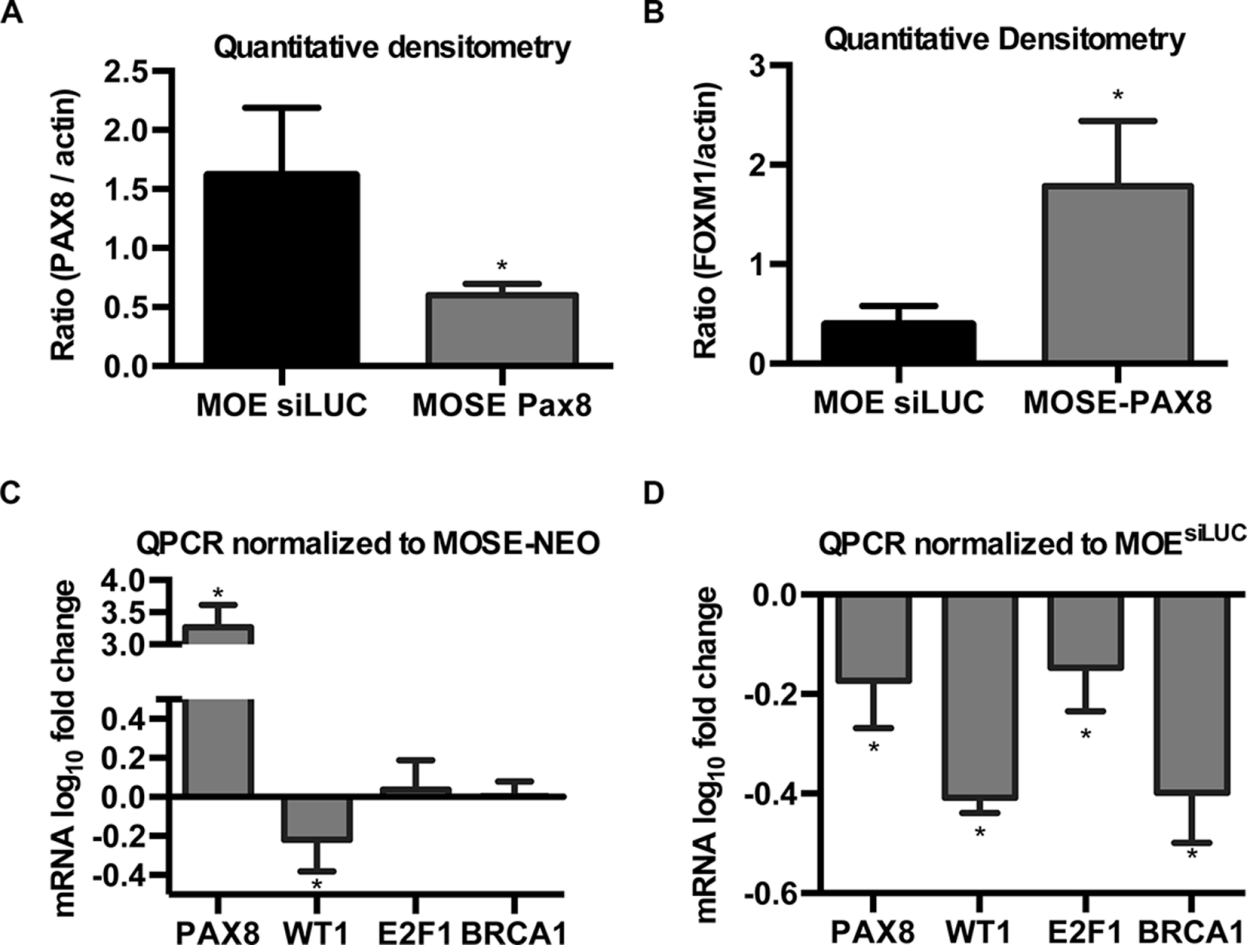
PAX8 has unique modes of action in the fallopian tube and ovary (**A**) Quantitative densitometry of western blots indicate forced expression of PAX8 in MOSE cells remains significantly lower than the endogenous levels in MOE (*n* = 3). (**B**) FOXM1 protein levels in MOSE-PAX8 are significantly higher than in MOE as demonstrated by quantification of western blots (*n* = 3). (**C**, **D**) mRNA levels (log_10_ fold change) of genes with known PAX8 binding sites in MOSE and MOE cells. Data was normalized to control cells (*n* = 3). Data represent mean ± SD. Significance is represented by * for *p* ≤ 0.05.

To verify that MOE cells express high levels of PAX8, and to demonstrate that the lack of functional effect after PAX8 silencing was not due to preexisting low levels of PAX8 protein, western blotting and quantitative densitometry were performed on MOE and MOSE-PAX8 cells. MOE cells expressed significantly higher levels of PAX8 even when compared to MOSE cells that have forced PAX8 expression from a constitutive CMV promoter (Figure 4A). Next, the endogenous expression levels of FOXM1 in MOE and MOSE cells were measured to examine whether the total level of FOXM1 was responsible for the difference between cell types. Quantitative densitometry confirmed that MOE cells expressed significantly less FOXM1 protein than MOSE-PAX8 (Figure 4B).

In addition to FOXM1, previous research has shown PAX8 can bind and transcriptionally activate WT1, E2F1, and BRCA1 [25–28]. qPCR was performed to determine if PAX8 differentially regulates these genes in the oviduct and the ovarian surface epithelium. In MOSE cells, PAX8 expression had no effect on E2F1 or BRCA1 (Figure 4C). In MOE cells, however, E2F1 and BRCA1 mRNA levels decreased after PAX8 silencing (Figure 4D). Forcing PAX8 expression in MOSE cells and silencing PAX8 expression in MOE cells both resulted in a decrease in WT1 mRNA (Figure 4C–4D). These findings suggest PAX8 has unique transcriptional activity in the oviduct and ovarian surface epithelium.

### Loss of PAX8 reduces FOXM1 in HGSC

**Figure 5:**
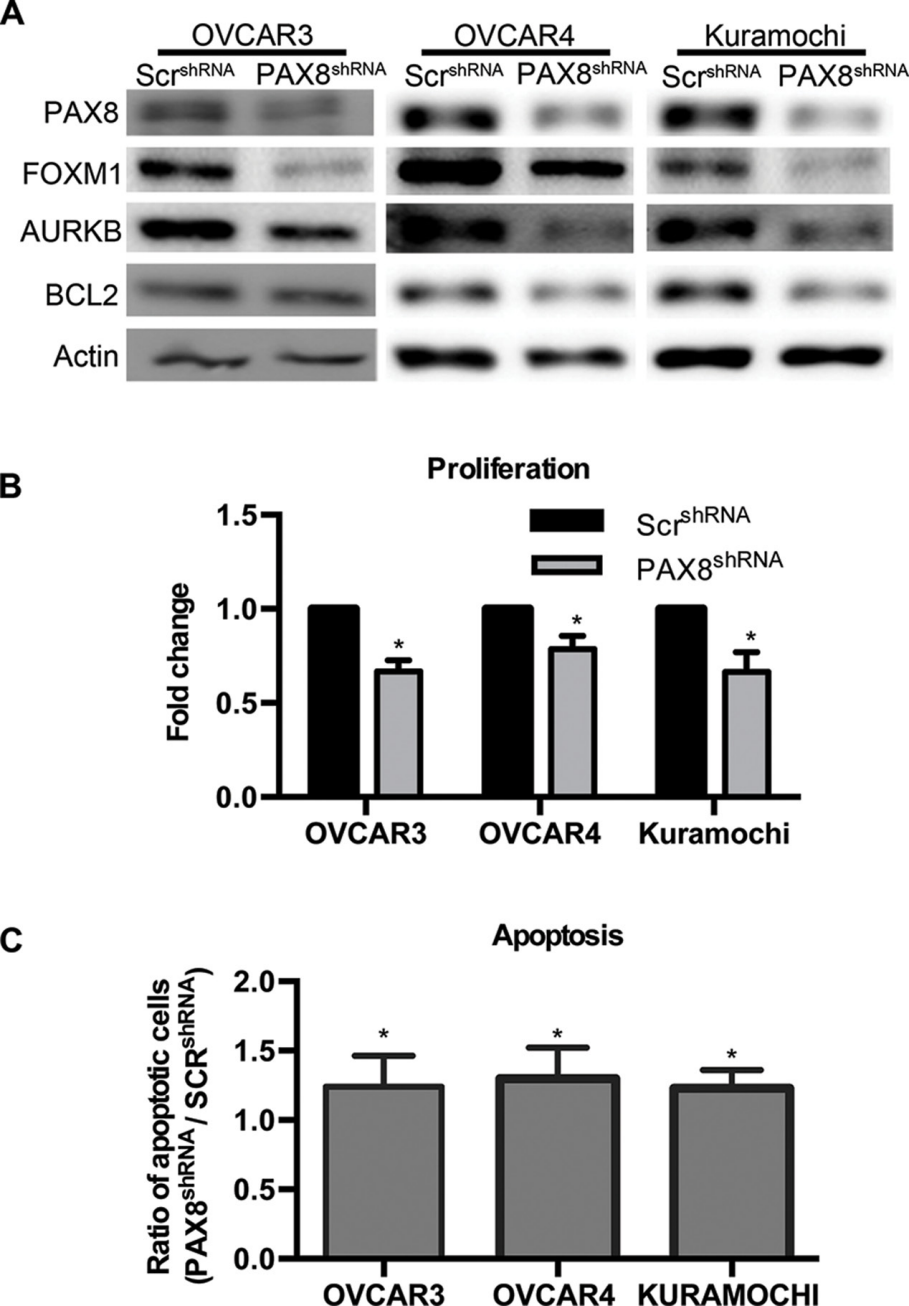
Loss of PAX8 reduces FOXM1 in HGSC (**A**) Representative western blots demonstrating a decrease in FOXM1, AURKB, and BCL2 after PAX8 knockdown in OVCAR3, OVCAR4, and Kuramochi. (**B**) Silencing PAX8 in HGSC significantly decreases proliferation (*n* = 3) after 72 hours. Data is represented as the fold change in proliferation from control. (**C**) Flow cytometry with Annexin V, APC and PI staining indicates an increase in apoptosis after PAX8 silencing in HGSC cells (*n* = 3). Data is represented as the ratio of total apoptotic cells compared to control.

To verify PAX8 expression in HGSC cell lines, western blotting was performed to compare PAX8 protein levels in Kuramochi, OVSAHO, OVCAR4, OVKATE, OVCAR3, OVCAR5, OVCA432 and SKOV3 (Supplementary Figure S2). The cell lines used in this study (OVCAR3, OVCAR4, and Kuramochi) expressed PAX8 at high levels. Reducing PAX8 levels in these cell lines resulted in decreased expression of FOXM1 and its downstream targets including AURKB (Figure 5A). There was also a decrease in the anti-apoptotic protein BCL2 after PAX8 knockdown (Figure 5A). BIRC5a and PLK were probed for by western blot but there was not enough protein for detection (data not shown). All three cell lines demonstrated a significant decrease in proliferation after PAX8 knockdown (Figure 5B). The mean log_10_ fold change in proliferation for OVCAR3 was 0.67, for OVCAR4 was 0.79 and for Kuramochi was 0.66 when compared to control. The decrease in proliferation seen in HGSC^siPAX8^ was greater than in the MOE^siPAX8^ cells where the mean log_10_ fold change was 0.88 compared to control. Based on previous reports that OVCAR3 undergoes apoptosis after PAX8 silencing and our data demonstrating a decrease in BCL2 after PAX8 knockdown, flow cytometry with Annexin V/PI staining was performed on OVCAR3, OVCAR4, and Kuramochi transfected with PAX8 shRNA. There was a slight, but significant increase in apoptosis after silencing PAX8 as compared to a negative control in all three cell lines (Figure 5C). These findings taken together suggest HGSC, if derived from normal fallopian tube cells, acquire changes that enhance reliance on PAX8 expression for survival. Alternatively, these results may be explained by the addition of oncogenic pathways when PAX8 is turned on in OSE cells.

## DISCUSSION

**Figure 6:**
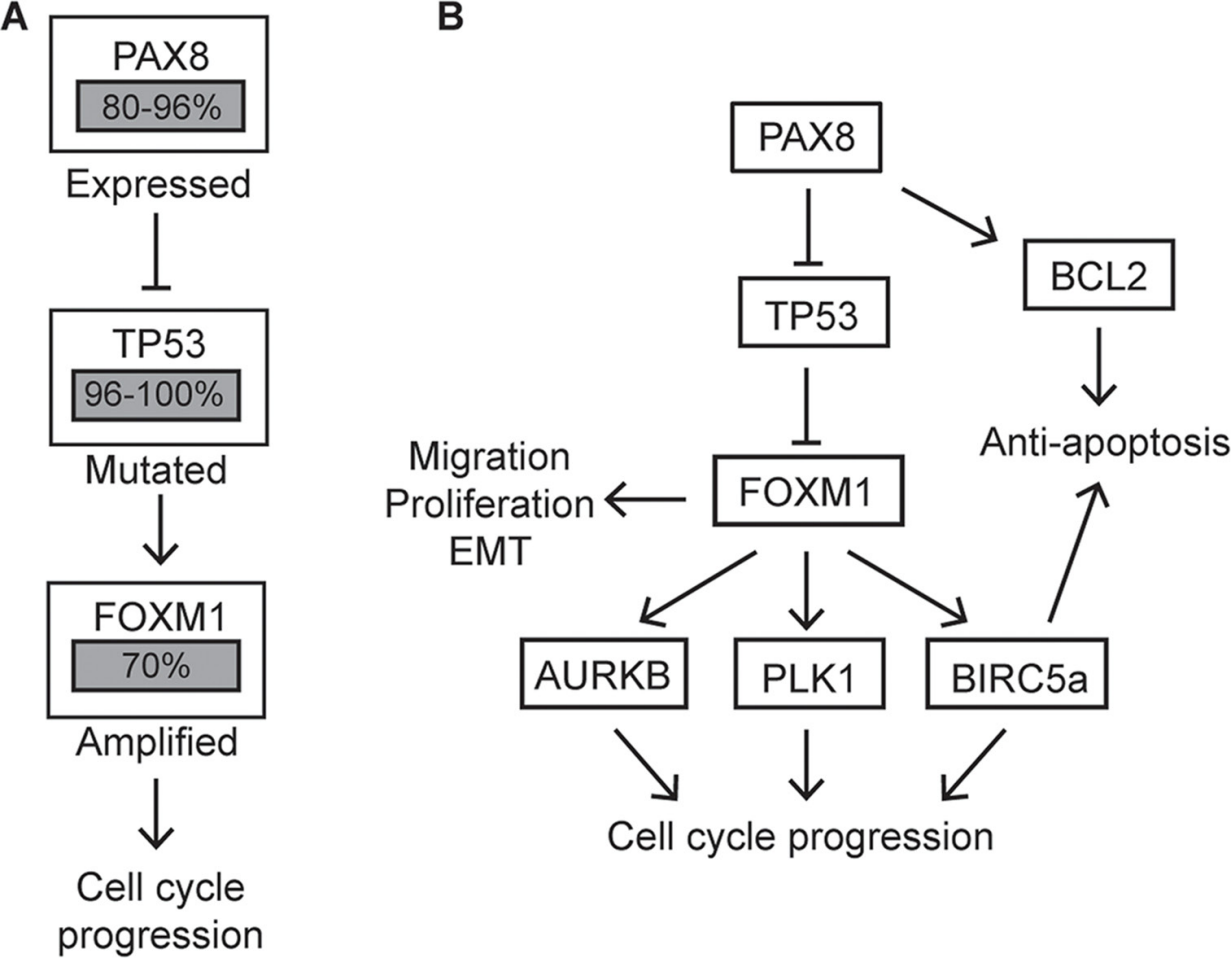
Altered pathways in HGSC compared to MOSE-PAX8 cells (**A)** Proposed role for PAX8 in amplifying the FOXM1 pathway in HGSC. Percentages of patient samples with alterations in these genes are highlighted in grey boxes. (**B)** A model of the effect forced PAX8 expression in MOSE cells has on the FOXM1 pathway.

This study explored the role of PAX8 in ovarian cancer progression by manipulating PAX8 expression and characterizing its effects in the fallopian tube, ovary, and HGSC cell lines. Several murine models of HGSC developed from the OSE acquire PAX8 expression, but its role in these cells had not been elucidated [13, 14]. Findings presented in this study demonstrate how PAX8 in MOSE cells, but not in MOE cells, can increase proliferation, migration and EMT. TCGA highlighted FOXM1 as one of the top altered genes in HGSC, with 87% of cases showing FOXM1 alteration [23]. FOXM1 is known to increase cell proliferation, migration, and EMT [29–31]. The findings presented here indicate upregulation of FOXM1 by PAX8 is at least partially responsible for the functional changes seen in MOSE and HGSC cells alteration of PAX8 expression. Since it has been shown that PAX8 can transcriptionally repress p53 and that p53 is a negative regulator of FOXM1 expression [21, 22], we hypothesize that PAX8 increases FOXM1 expression by inhibiting p53 activity (Figure 6A–6B).

In MOE cells where PAX8 is endogenously expressed, silencing PAX8 did not decrease expression of the FOXM1 pathway and there was only a slight decrease in proliferation in these cells. The loss of PAX8 in nontransformed oviductal cells did not impact migration, cell cycle, or apoptosis. Previous research has shown that FOXM1 expression is limited to dividing cells [32]. Compared to the OSE *in vivo*, the FTE cells are stable and only a small population (1–3%) are actively dividing [33]. This study shows that the FOXM1 levels in MOE are significantly lower than the levels seen in MOSE-PAX8. Therefore, PAX8 manipulation in MOE cells may have less functional changes than in MOSE and HGSC cells because the expression is much lower. The TCGA has also shown FOXM1 levels are lower in the fallopian tube compared to HGSC [23]. This is likely due to the fact that 96–100% of HGSC have a mutation in p53 that stabilizes the protein [23]. An increase in mutant p53 should cause an increase in FOXM1 regardless of cell of origin. This has important therapeutic implications because potential therapies that inhibit PAX8 expression might selectively target serous cancer cells without affecting normal fallopian tube or ovarian tissue.

Understanding the cell of origin of HGSC has vital importance for research and clinical treatment decisions. Current evidence suggests that fallopian tube serous tubal intraepithelial carcinomas (STICs) are associated with 20–60% of HGSC, but in up to 30% of HGSC cases there are no fallopian tube lesions, suggesting an ovarian origin [5]. If both the fallopian tube and the ovarian surface epithelium can serve as the progenitor cells for HGSC, then it is likely that the different cell types will have unique molecular pathways involved in malignant transformation. This study shows how genes with known functional binding sites for PAX8 such as E2F1, BRCA1, and WT1 are transcriptionally upregulated by PAX8 in the fallopian tube but not in the ovary. Yet despite these tissue specific roles for PAX8, the HGSC cell lines tested in this study uniformly showed a decrease in proliferation and an increase in apoptosis after PAX8 silencing. Further investigation into the molecular pathways affected by PAX8 silencing in HGSC cells could elucidate their cell of origin or demonstrate that this is an important target regardless of the cell of origin.

The future challenge is to examine MOSE-PAX8 and MOE^siPAX8^ cells on a genome wide scale to identify shared and distinct pathways in these cells controlled by PAX8. Since MOSE-PAX8 cells do not form tumors, it would be interesting to compare these cells with the molecular profile of malignant MOSE cells that form tumors [13]. In this way, researchers could identify pathways that interact with PAX8 in order for cells to become malignant. Research should also focus on ChIP-sequencing in MOSE and MOE cells to identify PAX8 binding sites and the direct regulatory roles of PAX8 in these cell types. Finally, since PAX8 can bind p53 and RB [25, 26], this study used murine cells that are not immortalized with SV40 and therefore have wildtype p53 and RB protein. While it was advantageous to use murine models to examine cells with wildtype p53, further research will be necessary to examine the role of PAX8 in human fallopian tube cells and ovarian surface epithelial cells.

In summary, this study reveals a distinctive role for PAX8 in the ovary compared to the fallopian tube that increases migration, proliferation, and EMT. These findings highlight the importance of deciphering a tumor's cell of origin because of the unique pathways that are deregulated depending on a tumor's progenitor cell. Yet despite the unique role of PAX8 in the ovary compared to the fallopian tube, these findings suggest HGSC cells are reliant on PAX8.

## MATERIALS AND METHODS

### Cell culture

All reagents were obtained from Life Technologies (Carlsbad, CA) unless otherwise indicated. Murine oviductal cells (MOE) and murine ovarian surface epithelial cells (MOSE) were obtained from Dr. Barbara Vanderhyden at the University of Ottawa. MOE and MOSE cells were cultured as previously described [34]. OVCAR4 cells were obtained from the National Cancer Institute from the Division of Cancer Treatment and Diagnosis Tumor Repository. Kuramochi cells were obtained from the Japanese Collection of Research Bioresources Cell Bank (JCRB). OVCAR4 and Kuramochi cells were cultured using RPMI 1640 media, supplemented with 10% FBS (Denville Scientific, Holliston, MA) and 1% pen/strep. OVCAR3 cells were purchased from the American Type Culture Collection (ATCC). OVCAR3 cells were cultured using MEM, supplemented with 20% FBS (Denville Scientific, Holliston, MA), 0.05 mg/mL Insulin, 1% non-essential amino acids, 1% sodium pyruvate, 1% L-glutamine, and 1% pen/strep. OVCAR3 and Kuramochi cells have been verified by STR analysis. The molecular profiles and *in-vivo* tumor growth capabilities of all three HGSC lines used in this study have been previously characterized [35–37].

### Stable cell lines and transient transfections

MOSE-PAX8 and MOSE-Neo stably transfected cell lines were created by transfecting MOSE cells with 10 μg of either mouse cDNA PAX8 plasmid (Transomic, Huntsville, AL, Catalog No. TCM1204) or empty vector with neomycin resistance as previously described [34]. Cells were plated at low density and treated with 0.15 mg/mL of G418 (Gemini bio-products, Woodland, CA). Individual stable clones were clonally selected and propagated using 0.05 mg/mL G418. Clones were verified for expression of PAX8 via immunoblotting and qPCR.

MOE cells were plated in 6 well plates at a volume of 100,000 cells per well 24 hours prior to transfection. A total of 400 ng/mL of PAX8 endoribonuclease small interfering RNA (siRNA) (Sigma-Aldrich, St. Louis, MO, EMU061581) was transfected into MOE cells using Mirus TransIT X2 (Mirus Bio LLC, Madison, WI) according to manufacturer's protocol. Cells were incubated for 72 hours in the presence of siRNA PAX8, and collected for protein analysis. An siRNA that decreases expression of the luciferase gene was used as a negative control (Sigma-Aldrich, St. Louis, MO, EHURLUC). Human PAX8 shRNA was purchased from Sigma-Aldrich (St. Louis, MO, TRCN0000021278), and transfected into OVCAR3, OVCAR4 and Kuramochi at 5 μg/mL for 72 hours, using TransIT X2 (Mirus Bio, Madison, WI) transfection reagent. A non-targeted shRNA was used as a control.

### Immunoblotting

Whole cell protein extract was obtained by collecting cells and lysing using RIPA buffer [50 mM Tris, pH 7.6, 150 mM NaCl, 1% Triton X-100, 0.1% SDS] with protease and phosphatase inhibitors (Sigma-Aldrich, St. Louis, MO). Protein concentration was determined using the BCA protein assay kit (Thermo Fisher Scientific, Waltham, MA). 20 ug of protein was loaded into 7–15% SDS-PAGE gels depending on the molecular weight of the desired protein and then transferred onto nitrocellulose membrane (Thermo Fisher Scientific, Waltham, MA). Membranes were blocked for 1 hour in 5% milk in Tris buffered saline Tween-20 (TBST) before overnight incubation at 4°C with primary antibody. The antibodies used include N-cadherin (Abcam, Cambridge, MA, ab12221, 1:300), Fibronectin (Sigma-Aldrich, St. Louis, MO, F3648, 1:1000), p53 (Santa Cruz Biotechnology, Dallas, TX, FL-393, 1:1000), FOXM1 (Santa Cruz Biotechnology, Dallas, TX, SC500, 1:200), PAX8 (Proteintech, Rosemont, IL, 10336-1-AP, 1:500). Antibodies from Cell Signaling (Beverly, MA) include AURKB (3094, 1:1000), PLK (4535, 1:1000), BCL2 (2876, 1:1000), and BIRC5a (2808, 1:500).

### Quantitative PCR

For mRNA analysis, cells were collected in 1 mL TRIzol (Life Technologies, Carlsbad, CA) per 1 × 10^5^ cells and isolated using chloroform separation, isopropanol precipitation and ethanol washing according to manufacture's protocol. RNA concentrations were determined using NanoVue plus spectrophotometer (GE healthcare, product code 28-9569-62). A total of 1 μg RNA was made into cDNA using iScript cDNA synthesis kit (Biorad, Hercules, CA). qPCR analysis was performed in a 96 well plate using Life Technologies ABI Viia7 machine. PCR reaction mixture was as follows: 5 μL FastStart SYBR green (2×) (Roche diagnostics, Indianapolis, IN), forward and reverse primers (0.5 μM), 2.6 μL DEPC water and 2 μL cDNA (2.5 ng/μL). PCR run protocol was 10min @ 95^°^C (hot start polymerase); 10 seconds @ 95^°^C followed by 30 seconds @ 60^°^C (40 cycles). All primers were validated for efficiency through serial dilutions and generation of a standard curve. Housekeeping genes included 18s rRNA and GAPDH. Fold change in mRNA expression was determined using the 2^ΔΔ^Ct method. Primers used are listed in Supplementary Table S1.

### Proliferation assays

Proliferation assays were performed as previously described [38]. Briefly, 1,000 cells per well were plated in a 96 well plate 24 hours before transfection. Cells were incubated for 0, 24, 48, and 72 hours before the assay was performed. To fix the cells, 20% tricholroacetic acid (TCA) was added to the plate and allowed to incubate for 1 hour at room temperature or several days at 4°C. After incubation the cells were washed 4 times with tap water and stained with 0.4% sulforhodamine B (SRB) in 1% acetic acid for 30 minutes. Extra SRB was removed by washing 4 times with 1% acetic acid. The plates were then allowed to fully dry before resuspending in 10 mM Tris buffer (pH 7). Absorbance was measured at 505 nm using a BioTek Synergy 2 microplate reader (BioTek, Winooski, VT). Absorbance was normalized to day 0 absorbance to determine the increase in proliferation.

### Migration assays

Wound closure assays were performed to measure migratory ability of cells. Cells were plated to 80% density in a 24 well plate to form a cell monolayer. A uniform wound was created through the cell monolayer using a p1000 pipette tip [39]. Pictures were taken at defined time points after wound creation using AmScope MU900 with Toupview software (AmScope, Irvine, CA). The area of the scratch was quantified using ImageJ NIH software. Percent of closure was determined by comparing the area of each time point to 0 hours.

The xCELLigence^®^ DP system (Acea Biosciences, San Diego, CA) was used to measure migration as previously described [34]. Approximately 4 × 10^4^ cells were resuspended in serum free media and plated in the upper chamber of a CIM plate 16. FBS was used as an attractant for cells to migrate through the CIM plate into the lower chamber. Measurements were taken every 15 minutes for 50 hours.

### Anchorage independent growth

Soft agar colony formation assay was performed as previously described [40]. Colonies were imaged on an AmScope MU900 microscope with Toupview software (AmScope, Irvine, CA). Image J NIH software was used to count colonies.

### Annexin V/PI apoptosis

Apoptosis assay using AnnexinV/PI staining was performed for HGSC and MOE cells. Cells were plated at 1000 cells/well in a 6 well plate. Cells were transfected for 72 hours as described above before collection. Cell media was centrifuged at 800 g to collect dead cells floating in the media. Live cells were collected using 0.25% trypsin, 1 mM EDTA (Invitrogen, Carlsbad, CA) to disrupt the cell monolayer. Total cell concentration of live and dead cells was approximately 1 × 10^6^ cells/ml. These cells were combined and resuspended in 300 ul 1X Annexin V Binding Buffer (140 mM NaCl, 4 mM KCl, 0.75 mM MgCl_2_ and 10 mM HEPES in DDW). 4 samples were created for each cell line: No staining, Annexin only staining, PI only staining, Annexin/PI staining. Propium iodide was added to cells to a final concentration of 2 ug/ml (Sigma-Aldrich, St. Louis, MO). Annexin V was diluted 1:60 and 1 ul was added to cells (Thermo Fisher Scientific, Waltham, MA). Cells were incubated in the dark for 15 minutes before adding another 400 ul Annexin V Binding Buffer. Cells were transferred to a cell strainer (Corning Incorporated, Corning, NY) and sent to the flow cytometry core at the University of Illinois-Chicago.

### Cell cycle analysis

Cell cycle analysis was performed for MOE cells. Cells were plated at a density of 25,000 cells/well in a 6 well plate. Cells were transfected for 72 hours as described above before collection using 0.25% trypsin (Invitrogen, Carlsbad, CA). Media and cells were centrifuged at 800 g to create a cell pellet. Cells were resuspended in 1 mL PBS and 1 mL 70% ethanol. Sample was centrifuged at 500 g and the media was decanted. Cell pellet was resuspended in 0.5 mL staining solution (2% RNase A, 2% propium iodide in PBS) and incubated for 30 minutes at room temperature in the dark. Cells were sent to the flow cytometry core at the University of Illinois-Chicago.

### Animals

All animal experiments were performed in accordance with the National Institutes of Health Guidelines for the Care and Use of Laboratory Animals and the established Institutional Animal Use and Care protocol at the University of Illinois at Chicago (UIC). In addition, the Animal Care Committee approved the protocol 11–066. Female NCr *nu/nu* athymic (nude) mice were obtained from Taconic (Hudson, NY, USA). Mice were housed in a temperature and light controlled environment (12 h light, 12 h dark) and provided food and water *ad libitum*.

The procedure of injections was performed as previously described [41]. Briefly, *i.p*. (1 × 10^7^ cells in PBS/animal) injections with MOSE-Neo or MOSE-PAX8 cells were performed on all mice (*n* = 6 mice/group). After 6 months, mice were euthanized by CO_2_ asphyxiation. Gross internal anatomy was inspected for tumors.

### Statistical analysis

Two-way ANOVA with Tukey's test multiple comparisons was performed when more than two groups were being analyzed. Paired student *t*-tests were performed for comparing two groups.

## SUPPLEMENTARY MATERIALS FIGURES AND TABLE


