# Supplementary material for: Loss of PAX8 in high-grade serous ovarian cancer reduces cell survival despite unique modes of action in the fallopian tube and ovarian surface epithelium
Source: Oncotarget. 2016 Apr 27;7(22):32785–95. doi: 10.18632/oncotarget.9051 (PMC5078051; doi:10.18632/oncotarget.9051)
Supplement: Supplementary file 1 [file oncotarget-07-32785-s001.pdf]

Loss of PAX8 in high-grade serous ovarian cancer reduces cell survival despite unique modes of action in the fallopian tube and ovarian surface epithelium

Supplementary Materials

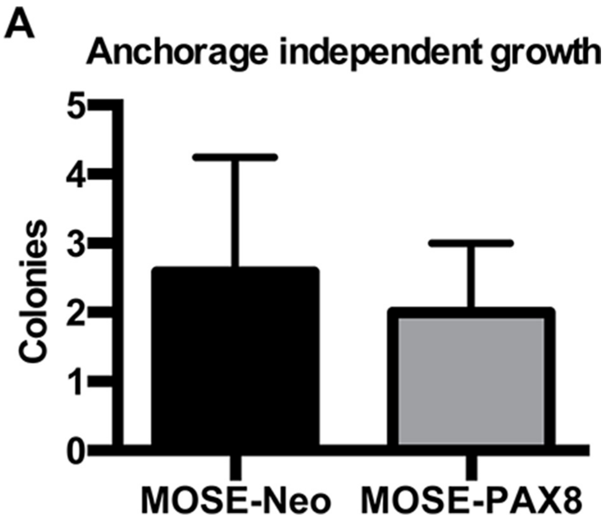

Supplementary Figure S1: Forced expression of PAX8 in the OSE does not induce neoplastic transformation. (**A**) Soft agar colony formation assay indicates no significant change in anchorage independent growth in MOSE-PAX8 cells compared to control after 14 days in culture ($n = 3$). Data represent mean ± SD.

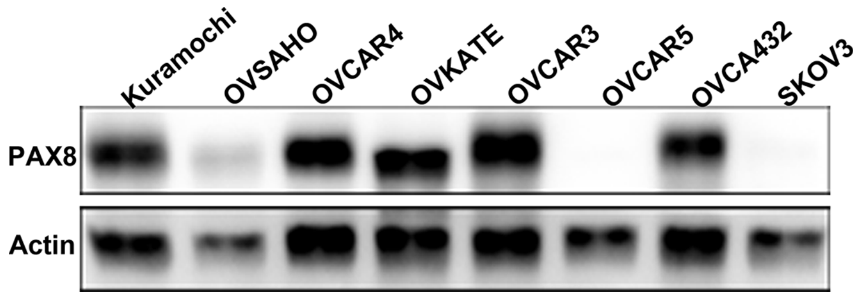

Supplementary Figure S2: HGSC cell lines used in this study express high levels of PAX8. (**A**) Representative western blots demonstrating PAX8 levels in the HGSC cell lines Kuramochi, OVSAHO, OVCAR4, OVKATE, OVCAR3, OVCAR5, OVCA432, and SKOV3.

Supplementary Table S1: List of gene names and primer probes used for QPCR analysis

Gene Name	Probe Name	Probe Sequence
PAX8	mPAX8 F<br>mPAX8 R	5′ – CGGCGATGCCTCACAACTCG -3′<br>5′ – CCGGATGCTGCCAGTCTCGT – 3′
FOXM1	mFOXM1 F<br>mFOXM1 R	5′ – TCCAGCTCTTGCAAATTTCCAGCC -3′<br>5′ – TTAGCGCTGTGATGATGATGCTCT – 3′
BIRC5a	mBIRC5a F<br>mBIRC5a R	5′ – GCAGCTGTACCTCAAGAACTA – 3′<br>5′ – GGTAGGGCAGTGGATGAAG – 3′
PLK1	mPLK1 F<br>mPLK1 R	5′ – CCACCTTAGTGACTTGCTACAG – 3′<br>5′ – CACTTGCTGACCCAGAAGAT – 3′
AURKB	mAURKB F<br>mAURKB R	5′ – CATCCCTGAGGAGGAAGACC – 3′<br>5′ – TTCATAGCAGAGCACCCCG – 3′
CCNB1	mCCNB1 F<br>mCCNB1 R	5′ – TCTGCACTTCCTCCGTAGA – 3′<br>5′ – GGAGAGCTCCATGAGGTATTTG -3′
CDC25B	mCDC25B F<br>mCDC25B R	5′ – TCCCTGTCATCTGAGTCCT – 3′<br>5′ – CGACTGGCTGCCTGAAT – 3′
WT1	mWT1 F<br>mWT1 R	5′ – CGGGTTTCCTCTTCTCCTTTG – 3′<br>5′ – CACATGCCCTGGCCTATAAAT – 3′
E2F1	mE2F1 F<br>mE2F1 R	5′ – GGTGATACCTTAAGTCCCTGTTC – 3′<br>5′ – CCCTCTCCCTTTCCCAATAAAT – 3′
BRCA1	mBRCA1 F<br>mBRCA1 R	5′ – CACAGCGTATGCCACAGAAA – 3′<br>5′ – ATCCTGGAGTTTGCATTG – 3′
